# Hidden in the Genome: The First Italian Family with North Carolina Macular Dystrophy Carrying a Novel *PRDM13* and *CCNC* Duplication

**DOI:** 10.3390/biomedicines13081904

**Published:** 2025-08-05

**Authors:** Beatrice Spedicati, Domizia Pasquetti, Aurora Santin, Stefania Zampieri, Anna Morgan, Stefania Lenarduzzi, Giuseppe Giovanni Nardone, Elisa Paccagnella, Stefania Cappellani, Laura Diplotti, Stefano Pensiero, Fulvio Parentin, Paolo Gasparini, Maurizio Battaglia Parodi, Giorgia Girotto

**Affiliations:** 1 Department of Medicine, Surgery and Health Sciences, University of Trieste, 34149 Trieste, Italy; beatrice.spedicati@burlo.trieste.it (B.S.); giuseppegiovanni.nardone@burlo.trieste.it (G.G.N.); paolo.gasparini@burlo.trieste.it (P.G.); giorgia.girotto@burlo.trieste.it (G.G.); 2 Institute for Maternal and Child Health—IRCCS “Burlo Garofolo”, 34137 Trieste, Italy; domizia.pasquetti@burlo.trieste.it (D.P.); stefania.zampieri@burlo.trieste.it (S.Z.); anna.morgan@burlo.trieste.it (A.M.); stefania.lenarduzzi@burlo.trieste.it (S.L.); elisa.paccagnella@burlo.trieste.it (E.P.); stefania.cappellani@burlo.trieste.it (S.C.); laura.diplotti@burlo.trieste.it (L.D.); stefano.pensiero@burlo.trieste.it (S.P.); fulvio.parentin@burlo.trieste.it (F.P.); 3 Ophthalmology Department, Vita-Salute San Raffaele University, 20132 Milan, Italy; battagliaparodi.maurizio@hsr.it

**Keywords:** inherited eye disorder, North Carolina macular dystrophy, whole exome sequencing

## Abstract

**Background:** North Carolina Macular Dystrophy (NCMD) is a non-progressive inherited macular dystrophy characterized by marked phenotypic variability. The genetic etiology of NCMD remains largely unknown, and only a limited number of families have been reported in Europe. **Methods**: We performed an in-depth investigation of an Italian family affected by NCMD using an integrated approach that combined SNP-array analysis, whole-exome sequencing, and long-read whole-genome sequencing. Additionally, we conducted a comprehensive review of NCMD-related literature. **Results:** We identified a novel 98 Kb duplication involving both *PRDM13* and *CCNC* genes in a three-generation kindred, where the proband exhibited severe macular alterations, while all other affected family members presented with a milder clinical phenotype. A review of the literature suggests different genotype–phenotype correlations and similar penetrance for duplications and single-nucleotide variants (SNVs) in described families. Specifically, smaller duplications may be associated with more severe phenotypes, while SNVs exhibit high phenotypic variability. **Conclusions**: In this study, we describe the first NCMD Italian family, in which the integration of second- and third-generation sequencing methods enabled the identification of a novel pathogenic *PRDM13* and *CCNC* duplication, thereby expanding the mutational spectrum of NCMD. Overall, these findings, together with the literature review, highlight the importance of selecting appropriate genetic testing approaches that allow the detection of non-coding variants and CNVs and thus enable accurate diagnosis and effective clinical management of patients and their families.

## 1. Introduction

Inherited retinal dystrophies are rare conditions characterized by a wide genetic heterogeneity, as, to date, more than 300 genes have been associated with these disorders, following all patterns of inheritance (primarily autosomal recessive, but also autosomal dominant, X-linked both dominant and recessive, and mitochondrial) [1,2]. From a clinical perspective, they comprise extensively diverse entities, such as rod-cone dystrophies, cone-rod dystrophies, chorioretinal degenerations, and macular dystrophies. The latter is a group of degenerative conditions that primarily affect the macula, with progressive macular atrophy that causes significant loss of the central vision [3]. Furthermore, psychophysical, electrophysiological, and histopathological changes can be highlighted also in the mid-peripheral and peripheral retina, thus confirming the presence of a widespread retinal involvement [4]. Additionally, these conditions are characterized by a broad inter- and intrafamilial clinical variability, which can further hamper the achievement of a genetic diagnosis, as it may foster the hypothesis of the presence of peculiar scenarios, such as dual molecular diagnoses. Indeed, the presence of two distinct genetic disorders segregating within the same individual or the same family is an increasingly recognized event that challenges the accuracy of genetic testing [5].

Among inherited macular dystrophies, a particularly rare condition is represented by North Carolina Macular Dystrophy (NCMD), a congenital, usually non-progressive disorder of the macula with unknown prevalence. The first NCMD family was described in 1971 by Lefler et al. [6], who reported a large kindred of seventy members, spanning four generations, living in North Carolina (USA) and descending from three Irish brothers who emigrated there in the 1830s [7]. Subsequent clinical evaluations of the affected family members were instrumental in defining the ophthalmological features of the condition [8,9]. After the first clinical report, further families have been described in a number of other countries around the world, including France, Belize, Germany, Korea, China, Georgia, Mexico, Turkey, and the United Kingdom [7,10,11,12,13,14,15,16,17].

From a genetic point of view, NCMD is an autosomal dominant condition that is fully penetrant but characterized by significant inter- and intrafamilial clinical variability [18]. Indeed, three different degrees of macular and retinal involvement have been described: patients may present few small yellow-white drusen-like lesions within the fovea (Grade I), larger confluent lesions (Grade II), or a wider involvement of the *fundus oculi* with severe central colobomatous-like chorioretinal atrophy that appears as a scar with a thick, white, fibrotic rim [19]. Patients with Grade I and Grade II lesions usually have no or mild central vision loss, while subjects with Grade III features show moderate visual impairment. Despite being an uncommon finding, visual acuity may be further hindered by the development of choroidal neovascularization; color vision evaluation, full-field electroretinography, and electro-oculography results are usually within normal ranges [20,21].

The genetic bases of NCMD have long remained unclear, and more than forty years have passed between the first published clinical description of the disease [6] and the identification of its genetic determinants. Linkage analyses performed in large families in the 1990s and 2000s allowed to firstly pinpoint the *MCDR1* locus on chromosome 6q16 [11,22]. Additional studies highlighted how two additional loci are involved in the pathogenesis of NCMD, namely *MCDR2* on chromosome 4p15.32 [23] and *MCDR3* on chromosome 5p15.32 [24]. Sequencing analyses of all three critical regions proved successful only for the study of the *MCDR2* locus, as heterozygous variants within the *PROM1* gene have been causatively linked with the condition [25]. Conversely, no causative coding variants within *MCDR1* or *MCDR3* have been identified.

The advent of high-throughput sequencing technologies allowed us to go beyond the analysis of exonic regions, and in 2016 Small et al. [26] described the first peculiar genomic alterations within the *MCDR1* and *MCDR3* loci. Specifically, concerning *MCDR1*, three single nucleotide variants within an intergenic region corresponding to a DNase I hypersensitivity site (DHS) upstream of the *PRDM13* and *CCNC* genes and a complete duplication of the *PRDM13* gene were identified. Regarding *MCDR3*, a duplication of the entire *IRX1* gene was highlighted through Whole Genome Sequencing (WGS) analysis. Collectively, these findings suggested that NCMD is due to the dysregulation of gene expression rather than to the disruption of the coding sequence of the associated genes.

Herein, we report the clinical and genetic data of the first Italian NCMD family, contributing to further dissect the intrafamilial clinical variability that characterizes this condition and providing a comprehensive literature review.

## 2. Materials and Methods

### 2.1. Ethical Statement

All the analyses were performed following the relevant guidelines and regulations. Written informed consent was obtained from all participants or, in the case of underage patients, their legal guardians. The study was conducted in accordance with the tenets of the Helsinki Declaration.

### 2.2. Patient Enrollment and Clinical Evaluation

The proband and her family were referred for genetic counselling to the Medical Genetics Unit of the IRCCS “Burlo Garofolo” (Trieste, Italy). During the genetic visit, a detailed personal and familial anamnesis was obtained, and a dysmorphological examination was performed.

A complete ophthalmological assessment was performed, including best-corrected visual acuity (BCVA), Hirschberg corneal light reflex test, Cover test, Lang I and II stereo test, ocular motility examination, Goldmann intraocular pressure, cycloplegic refractions, slit-lamp biomicroscopy and dilated-fundus examinations, electroretinography (ERG), visual evoked potentials (VEP), optical coherence tomography (OCT), and optical coherence tomography angiography (OCTA).

### 2.3. DNA Extraction and Quality Control

Genomic DNA was extracted from peripheral whole-blood samples of the patient, her parents, and other family members using the QIAsymphony® SP instrument with the QIAsymphony® Certal Kit (Qiagen, Venlo, The Netherlands), following the manufacturer’s instructions. DNA quality and concentration were assessed with the Nanodrop ND 1000 spectrophotometer (NanoDrop Technologies Inc., Wilmington, DE, USA).

### 2.4. Whole Exome Sequencing (WES) Analysis

Trio WES analysis was performed on an Illumina NextSeq 550 Instrument (Illumina Inc., San Diego, CA, USA); genomic libraries were prepared using the Twist Human Exome 2.0 Plus Comprehensive Exome Spike-in kit (Twist Bioscience, San Francisco, CA, USA), according to the manufacturer’s protocol. WES protocol and secondary and tertiary analyses were carried out as already reported [27]. Variant filtering was based on the clinical features of the patient described through Human Phenotype Ontology (HPO) terms (https://hpo.jax.org/). Variant frequency was verified in gnomAD (https://gnomad.broadinstitute.org/). The deleteriousness of all identified variants was assessed through in silico prediction tools [28,29,30,31,32]. Pathogenicity of already-reported variants was assessed through ClinVar (https://www.ncbi.nlm.nih.gov/clinvar/) and The Human Gene Mutation Database (HGMD) (https://my.qiagendigitalinsights.com/bbp/view/hgmd/pro/start.php). WES protocol also allowed the detection of copy number variants (CNVs), which were classified and prioritized using public databases such as Clinvar, dbVAR (https://www.ncbi.nlm.nih.gov/dbvar/), and Decipher (https://www.deciphergenomics.org/). All databases were last accessed on 3 March 2025.

### 2.5. SNP-Array Analysis

SNP-array analysis was performed using the Human OmniExpress Exome-8 Bead v1.6 Chip (Illumina Inc., San Diego, CA, USA) containing 960,919 loci derived from phases I, II, and III of the International HapMap project. The array includes over 274,000 functional exonic markers, offering unprecedented coverage of putative functional exonic variants selected from 12,000 whole exome and genome sequences.

Genomic DNA for each sample was processed according to Illumina’s Infinium HD Assay Super protocol. Raw image intensity data normalization, genotype clustering, and genotype calling of individual samples were performed using Illumina’s Genome Studio v2.0.5 software (cnvpartition 3.2.1). Copy number variations (CNVs) were mapped to the hg19 human reference genome and annotated with UCSC RefGene. Allele detection and genotype calling were performed with Genome Studio software, and data analysis was performed with NxClinical software version 6.1 (BioDiscovery, Inc., El Segundo, CA, USA).

The significance of each detected CNV was determined by comparing all chromosomal alterations identified in the patient with those collected in an in-house database of ~6500 patients studied with SNP-arrays since 2020 and in public databases, including the Database of Genomic Variants (DGV), DECIPHER, and ClinGen. CNVs considered polymorphic in the DGV database were not considered in the interpretation of the results.

### 2.6. PromethION

CNV genomic breakpoints were further characterized using Nanopore technology (PromethION 24, Oxford Nanopore). Briefly, high-molecular-weight DNA was extracted, and libraries were prepared using the ligation sequencing kit, followed by sequencing on the PromethION platform, according to the manufacturer’s instructions. A sequencing run was performed selecting the high-accuracy basecalling model, and resulting reads were trimmed using NanoFilt [33], using the following parameters: “−q 10-l 500 -headcrop 50”. Clean FastQ data were then aligned to the GRCh38 reference genome using minimap2 [34]. Structural variants were identified with Sniffles2 [35]. Visualizations of the detected variants were generated using Samplot [36].

### 2.7. Literature Review

A literature review of articles published before April 2025 was performed using PubMed, and papers containing the keywords “North Carolina Macular Dystrophy” and “genetics” were retained. All cases reported in the literature were collected, and a comparison of both the clinical and molecular features was carried out.

## 3. Results

### 3.1. Case Report

The proband is an eight-year-old Italian girl who was referred to genetic counseling with a clinical diagnosis of macular atrophy.

The patient was initially referred to the ophthalmology clinic at nine months of age due to an abnormal red reflex: the examination revealed a wide bilateral area of chorio-retinal atrophy involving the macular region. Molecular testing for cone dystrophies was carried out and did not identify any causative variant. Ophthalmological follow-up in the subsequent years highlighted a stable *fundus oculi* picture.

**Figure 1:**
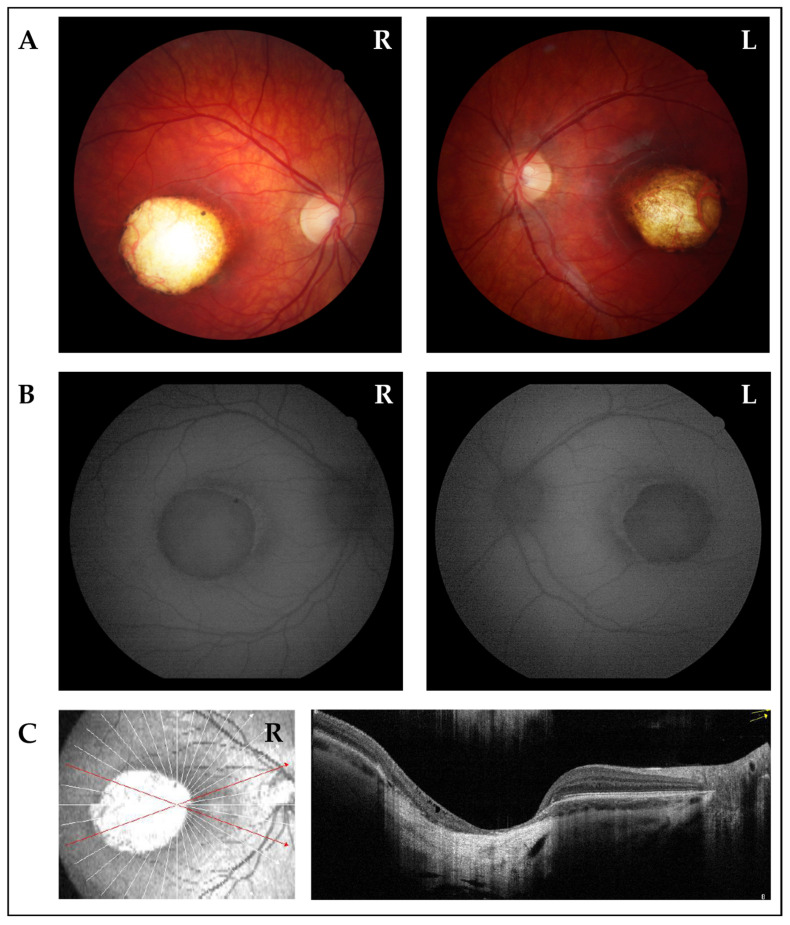
Ophthalmological imaging studies of the proband. (**A**) Fundus photographs of right eye (R) and left eye (L) show wide colobomatous-like chorio-retinal atrophic macular lesions and visible sclera. (**B**) Fundus autofluorescence of right (R) and left (L) eyes displays the absence of fluorescence in macular lesions, indicating the absence of retinal pigment epithelium (RPE). The edge of the lesions exhibited mild high-fluorescence halo. (**C**) OCT scan of the right eye (R) displays a wide area characterized by atrophy of neurosensory retina, RPE, and choroid, with underlying increase in backscattering signal. Focal subretinal hyperreflectivity is observed at the edge of the lesion.

At eight years of age the patient was referred to the Ophthalmological Clinic of the Institute for Maternal and Child Health I.R.C.C.S. “Burlo Garofolo” (Trieste, Italy), where a re-evaluation was performed. BCVA was 20/100 in the right eye (RE) and 20/50 in the left eye (LE) with mild anisometropia (cycloplegic refraction −0.75 D sphere +0.25 cyl RE and +1.75 D sphere +1.25 cyl LE). Orthoptic assessment revealed exophoria and convergence deficit. Intraocular pressure (IOP) and biomicroscopy findings from the anterior segment were unremarkable, while fundus examination confirmed bilaterally a wide area of chorio-retinal atrophy involving the macular region, as shown in Figure 1, and pigmentary dots in the mid-peripheral retina. RE *fundus* additionally presented a smaller area of chorio-retinal atrophy in the inferotemporal quadrant. Macular OCT and OCTA revealed bilaterally a wide area characterized by atrophy of the neurosensory retina, retinal pigment epithelium (RPE), and choroid, with an underlying increase in backscattering signal. Focal subretinal hyperreflectivity was observed at the edge of the lesion. Choroidal neovascularization was not detectable in either eye. This peculiar ophthalmological picture led to a clinical diagnosis of Grade III NCMD. Retinal function was also assessed through ERG, which revealed amplitude asymmetry in the rod response (OD: 66 μV; OS: 173 μV) and marked asymmetry in the cone response (OD: latency 22 ms, amplitude 48 μV; OS: 20 ms, 225 μV). VEP demonstrated bilaterally detectable responses to flash stimulation, with prolonged P2 wave latencies. Overall, these findings are consistent with rod–cone dysfunction, more pronounced in the right eye (OD), involving both photopic and scotopic pathways.

**Figure 2:**
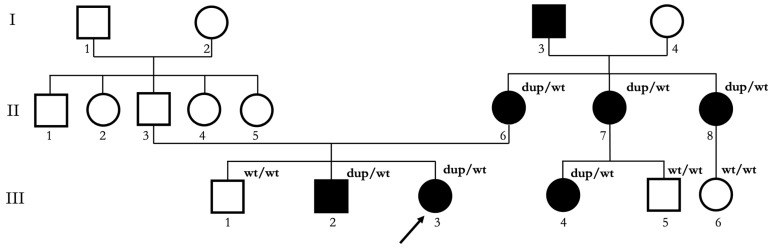
Pedigree of the Italian NCMD family described in this study. Squares indicate male subjects; circles indicate female subjects. Proband is indicated with a black arrow. Black filling indicates affected subjects. Roman numerals indicate different generations. Arabic numerals indicate different subjects in each generation. dup/wt indicates subjects who underwent genetic testing and resulted carriers of the 6q16.2 microduplication. wt/wt indicates subjects who underwent genetic testing and did not result carriers of the 6q16.2 microduplication.

Upon genetic counseling, a complete family history was collected. The family has a multi-generational Italian ancestry, with lineage traced back at least three generations within Italy. As shown in Figure 2, the proband (III:3) had a 16-year-old healthy brother (III:1) and a 12-year-old brother (III:2) that was reported to have a clinical diagnosis of Grade I NCMD. The siblings’ mother (II:6), her two sisters (II:7, II:8), and the daughter (III:4) of one of the sisters were also affected by NCMD.

**Figure 3:**
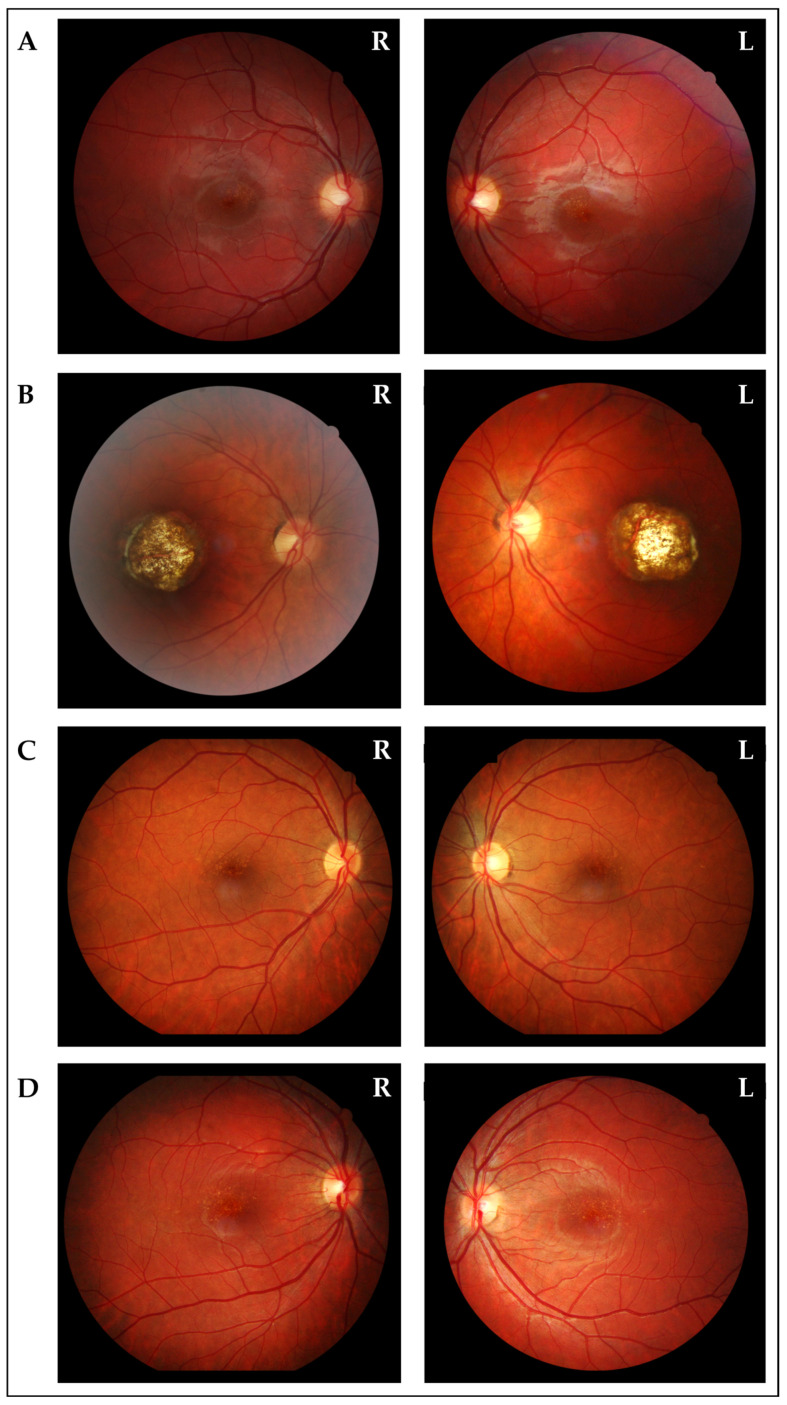
*Fundus oculi* photographs of the other affected members. (**A)** *Fundus oculi* of the brother (III:2), showing bilateral diffuse, small, orange–yellow, confluent, drusen-like lesions and tenuous pigmentary changes in the macular region. (**B**) *Fundus oculi* of the mother (II:6), showing bilaterally a wide area of chorio-retinal atrophy and pigmentary changes involving the macular region. (**C**) *Fundus oculi* of one of the aunts (II:7), showing bilateral diffuse, small, orange–yellow, confluent, drusen-like lesions and tenuous pigmentary changes in the macular region. (**D**) *Fundus oculi* of the proband’s cousin (III:4), showing bilateral diffuse, small, orange–yellow, confluent, drusen-like lesions and tenuous pigmentary changes in the macular region.

Upon ophthalmological examination, the proband’s mother (II:6) presented with BCVA of 20/50 in the RE and 20/40 in the LE with mild myopia (cycloplegic refraction -1.25D sphere −0.25 cyl RE and −0.75 D sphere −0.50 cyl LE). Orthoptic assessment, IOP, and biomicroscopy findings from the anterior segment were unremarkable; *fundus oculi* examination revealed bilaterally a wide area of chorio-retinal atrophy and pigmentary changes involving the macular region (Figure 3). RE *fundus* additionally presented a small choroidal nevus in the superotemporal quadrant. Macular OCT and OCTA revealed bilaterally a wide area characterized by atrophy of the neurosensory retina, retinal pigment epithelium (RPE), and choroid, with an underlying increase in backscattering signal. Focal subretinal hyperreflectivity was observed at the edge of the lesion. Choroidal neovascularization was not present in either eye.

*Fundus oculi* evaluation of the 12-year-old brother (III:2), the proband’s aunts (II:7, II:8), and the proband’s cousin (III:4) showed diffuse, small, orange–yellow, confluent, drusen-like lesions and tenuous pigmentary changes in the macular region, whereas BCVA, orthoptic assessment, IOP, and biomicroscopy findings from the anterior segment, OCT, and OCTA were unremarkable.

Finally, the proband’s maternal grandfather (I:3) was also reported to present NCMD, but his ophthalmological reports were not available for review.

Trio WES analysis was performed on the proband, her affected mother, and her healthy father. No pathogenic Single Nucleotide Variants within genes known to be associated with inherited retinal dystrophies were identified. CNV analysis allowed us to hypothesize the presence of a duplication of approximately 68.5 Kb in the 6q16.2 chromosomal region, including both the *PRDM13* (MIM: * 616741) and *CCNC* (MIM: * 123838) genes. Data analysis suggested the presence of the duplication in both the proband’s and her mother’s DNA.

**Figure 4:**
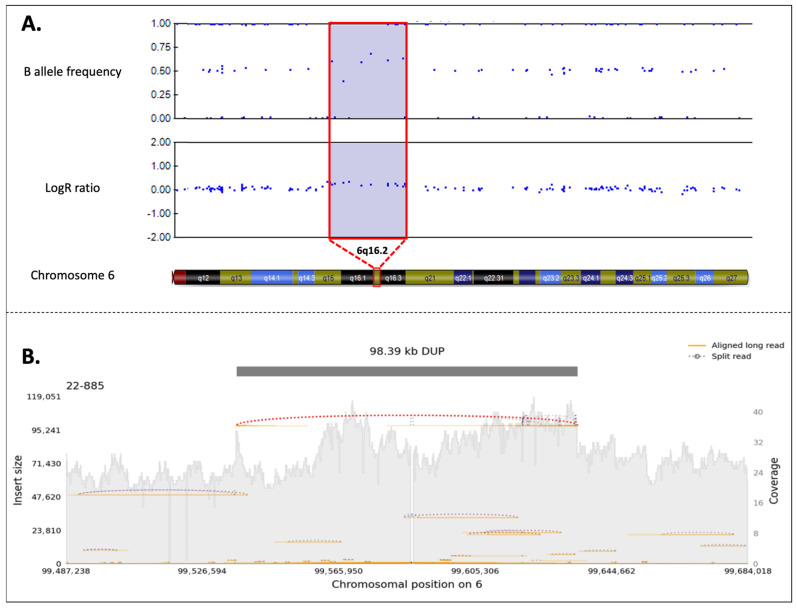
(**A**) SNP-array analysis of the reported case. The figure is a schematic representation of the duplication (copy number variant (CNV = 3) in the 6q16.2 region, highlighted in violet. In the duplication region, it is possible to observe the presence of four B-allele frequencies (BAF), and the increase in Log R ratio (LRR) from baseline results from the increase in the intensity signal intensity of a copy number-expanding region (about +0.6) (duplication or amplification). The BAF is a measure of the proportion of alleles at a locus. In a normal diploid region, BAF values typically form three clusters. However, in the presence of a duplication, an additional BAF cluster emerges due to the increased number of alleles. The LRR quantifies the normalized intensity of the probe signal corresponding to each genomic position. An elevated LRR value, in this case approximately +0.6, indicates an increase in DNA dosage consistent with duplication. This duplication includes the *PRDM13* and *CCNC* genes. (**B**) PromethION identification of the duplication. After PromethION sequencing, the 98.39 Kb duplication involving *PRDM13* and *CCNC* genes was confirmed, with breakpoint mapping at chr6:99,536,433-99,634,823 (GRCh38). The gray shaded area represents the depth of coverage across the region (also known as sequencing coverage, which refers to the number of times a given region of DNA is sequenced). It is expected to be higher in the presence of a duplication. Values of the coverage are specified in the right y-axis. Individual long-read alignments (i.e., the mapping of each long sequencing read to the reference genome) are shown as orange lines, in contrast with split reads (i.e., reads partially aligned to the reference genome due to the presence of a structural variation), which support the duplication and are shown as dotted lines. Insert size is plotted on the left y-axis, and chromosomal position is indicated on the x-axis.

The duplication was confirmed through SNP-array analysis and nanopore sequencing, which allowed us to better define the size (98.39 Kb) and the breakpoints of the genomic rearrangement (NC_000006.12:g.99536433_99634823dup) (Figure 4A and 4B). Segregation analysis was performed on the proband’s healthy brother (III:1), resulting absent, and her affected brother (III:2), where its presence was confirmed. It was also possible to extend the analysis to one of the affected sisters (II:7) of the mother’s proband and her two children (III:4, III:5): the woman and her daughter resulted positive, while the son resulted negative, as expected.

### 3.2. Literature Review

**Figure 5:**
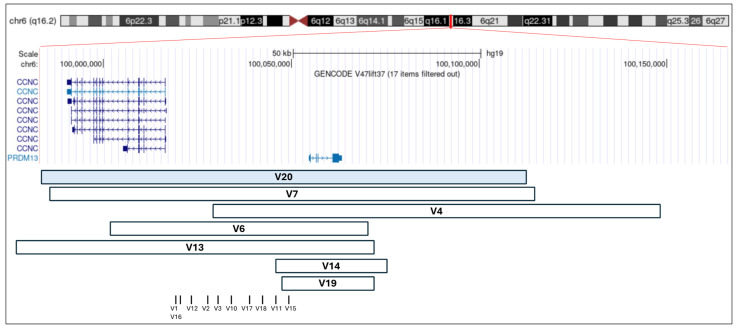
Schematic representation of reported duplications involving *PRDM13* and *CCNC*, and SNVs. Each square represents a previously reported duplication, and each vertical bar corresponds to a previously reported SNV (see Table 1). The V20 duplication, identified in the family described in this study, is highlighted in light blue. The genomic coordinates refer to the GRCh37/hg19 assembly and were visualized using the UCSC Genome Browser.

To date, 17 distinct *MCDR1*-linked variants, including the one reported here as V20, have been described in 32 families. Of these, ten are single-nucleotide variants (SNVs), all affecting an intergenic regulatory region corresponding to a DNase I hypersensitivity site (DHS6S1, OMIM 616842, 6q16.2), located upstream of two oppositely transcribed genes, *PRDM13* and *CCNC*. In the remaining cases, seven different tandem duplications, variably involving the two genes, have been reported. Molecular details of reported variants are described in Table 1 and represented in Figure 5.

**Table 1:** Reported *MCDR1*-linked variant, referred to GRCh37/h19 assembly. ‘Affected’ refers to patients carrying the molecular defect; ‘Unaffected’ refers to healthy relatives in whom genetic testing did not reveal the presence of the defect.

Variant Type	Variant Number	GRCh37/h19	References	Number of Families	Affected Individuals	Unaffected Individuals	Intrafamiliar Variability
**Tandem** **Duplication**	V20	chr6:99983358-100082211dup	Present study	1	5	2	Yes
	V7	chr6:g.99984309-100082698dup	[37]	2	10	9	Yes
	V4	chr6:g.100020205-100143306dup	[26]	1	11	4	Yes
	V6	chr6:g.99996226-100065137dup	[38]	1	6	5	Yes
	V13	chr6:g.99932464-100067110dup	[39]	1	3	0	Yes
	V14	chr6:g.100008141-100064368dup	[17]	1	6	2	No
	V19	chr6:100019429-100167607dup	[16]	1	7	0	No
**SNV**	V1	chr6:g.100040906G>T	[26]	6	60	29	Yes
			[20]	1	3	0	Yes
	V2	chr6:g.100040987G>C	[26]	3	13	5	Yes
			[18]	3	6	0	Yes
			[40]	1	2	1	Yes
	V3	chr6:g.100041040C>T	[26]	1	2	0	NA
	V10	chr6:g.100046783A>C	[41]	1	2	0	NA
			[42]	1	5	0	Yes
	V11	chr6:g.100046804T>C	[41]	2	6	0	Yes
	V12	chr6:g.100040974A>C	[14]	1	6	2	Yes
	V15	chr6:g.100046940A>G	[20]	1	4	1	Yes
	V16	chr6:g.100040906G>C	[20]	1	3	3	Yes
	V17	chr6:100046790T>C	[43]	1	4	1	Yes
	V18	chr6:100046802G>A	[43]	1	3	0	No

## 4. Discussion

Very few NCMD families of European ancestry have been reported so far in the scientific literature [7,10], and, to our knowledge, our study presents the first Italian family affected by this rare macular disorder. Specifically, we have provided a detailed clinical and genetic characterization of three-generation kindred carrying a novel duplication of approximately 98 Kb in the 6q16.2 chromosomal region, involving both *PRDM13* and *CCNC* genes.

*PRDM13* encodes a transcription factor involved in retinal neuronal differentiation, and its temporal expression pattern, initially sustained and subsequently downregulated during or after retinal cell differentiation, is essential for proper retinal development [44]. In mice, *Prdm13* expression begins at embryonic day 12.5 in the neuroblastic layer, while single-cell RNA sequencing data from the human embryonic retina indicate peak *PRDM13* expression between days 67 and 80, coinciding with the emergence of amacrine cells and the initiation of their synaptic connections with retinal ganglion cells [45]. Indeed, Prdm13 plays a critical role in the differentiation of specific subsets of amacrine cells and their synaptic connections [46]. While *PRDM13* expression is restricted to the retina, *CCNC*, which encodes a cell cycle regulator, is ubiquitously expressed. The precise pathogenic mechanism by which intronic variants or tandem duplications, affecting the non-coding region between the two genes, lead to disease remains largely unclear. However, recent genome-wide multi-omics studies, coupled with *in vitro* and *in vivo* enhancer assays, suggest that SNVs within the *PRDM13* locus disrupt cis-regulatory elements, non-coding DNA sequences that regulate the transcription of neighboring genes. These findings support a model in which NCMD results from aberrantly elevated *PRDM13* expression, potentially due to an insufficient downregulation during retinal differentiation [20].

To date, ten non-coding SNVs and seven tandem duplications, variably involving *CNCC*, have been reported as causative of NCMD. Our literature review focuses on exploring intrafamilial phenotypic variability in NCMD, defined as different degrees of macular involvement among patients carrying the same molecular defect. Regarding tandem duplications, a two-generation Turkish family carrying a 56.2 kb tandem duplication (V14) and lacking phenotypic variability was described by Small et al. [17]. Similarly, Chamaco et al. reported a Mexican family with seven affected individuals, all carrying a 148 kb duplication (V19) and presenting with a comparable severe phenotype, characterized by symmetric, excavated, coloboma-like chorioretinal macular lesions [16]. These two families shared a similar molecular defect, consisting of relatively small duplications involving only *PRDM13*. Conversely, duplications involving both *PRDM13* and *CCNC*, including the one reported here for the first time (V20), are associated with marked phenotypic variability. Consistently, intrafamilial clinical variability was also evident in our case, as the proband presented with grade III NCMD, while all other affected members exhibited a milder phenotype. Therefore, it can be speculated that smaller duplications may lead to a stronger dysregulation of the physiological temporal expression of *PRDM13*. However, these clinical observations require confirmation through clinical studies involving larger cohorts and functional validation.

Regarding SNVs, intrafamilial phenotypic variability has been described in all reported families with the exception of the one characterized by Seo et al. [43]. Indeed, in this Korean family, all affected individuals exhibited Grade I NCMD, characterized by confluent drusen at the fovea and preserved visual acuity. Therefore, while it is not currently possible to establish a definitive genotype-phenotype correlation for non-coding SNVs, our literature review confirms that phenotypic variability remains a key feature of the condition, particularly when caused by SNVs, and must be carefully considered during genetic counselling for NCMD. The marked intrafamilial clinical variability in the reported family could have been, at first sight, extremely misleading, leading to the hypothesis of the presence of a dual molecular diagnosis [5]. However, in this case, the integration of different technologies was instrumental in the achievement of the correct genetic diagnosis.

Similarly to phenotypic variability, incomplete penetrance may complicate genetic counselling. Our literature review showed that, when performed, co-segregation between the molecular defect and some degree of NCMD is consistently observed, including in our family, supporting the hypothesis that the disease is highly penetrant. However, these observations lead to several considerations, including the possibility that Grade I disease may remain asymptomatic and therefore undiagnosed unless actively investigated through detailed ophthalmologic examination. Conversely, NCMD clinical presentation may be mimicked by phenocopies, such as drusen of age-related macular degeneration, Stargardt macular dystrophy, Best macular dystrophy, torpedo maculopathy, and toxoplasmosis [47]. Therefore, clinical suspicion of NCMD requires a comprehensive clinical and molecular assessment to precisely define the macular involvement and exclude alternative etiologies or dual diagnoses in the proband. In addition, segregation analysis of the familial variant, always coupled with ophthalmologic examination, is recommended in at-risk relatives.

All these considerations highlight the importance of the appropriate choice of genetic testing when addressing conditions characterized by a wide clinical and genetic heterogeneity. Specifically, while WES presents considerable limitations in detecting CNVs and SNVs in regulatory regions, alternative approaches, referred to as third-generation sequencing, have been developed in recent years to overcome these challenges. Indeed, long-read sequencing has not only enabled the identification of variants in non-coding regions but also improved the detection of structural variants, including CNVs, enhancing the ability to accurately define their size and breakpoints [48].

While extending the analysis beyond coding regions increases diagnostic yield and enables genetic diagnoses for a greater number of individuals [49], we are still far from fully understanding the impact of CNVs in non-coding regions on clinical phenotypes. Regardless of the accuracy of current sequencing technologies, such variants are often classified as Variants of Uncertain Significance, primarily due to the difficulty in predicting their biological consequences [49]. In this context, functional studies are frequently required to assess the effects of regulatory region variants on splicing, transcription, RNA processing, and stability, as well as chromatin architecture. Notably, the integration of genomic and functional approaches has recently proven to be a successful strategy in demonstrating the pathogenicity of non-coding variants in several conditions beyond NCMD, including Charcot-Marie-Tooth disease [50], Stargardt disease [51], and various neurodevelopmental disorders [52,53].

Altogether, we report a successful example of the integration of second- and third-generation sequencing methods, which collectively enabled the characterization and clinical correlation of a novel *PRDM13* and *CCNC* duplication, thereby expanding the mutational spectrum of NCMD. These findings highlight the importance of investigating non-coding regions in NCMD and functionally assessing how CNVs can be disease-causing in order to ensure an accurate molecular definition. While no therapies currently address the underlying genetic defect in NCMD, establishing a molecular diagnosis enables individualized clinical management. This includes the initiation of surveillance for choroidal neovascularization, a potential and treatable complication of the disorder [54], and ensures patient eligibility for future clinical trial participation. Additionally, molecular diagnosis provides important benefits for families by informing on recurrence risk for probands and enabling cascade genetic testing in at-risk relatives.

## Data Availability

The original data presented in the study are openly available in the main text.

## References

[1] Kao H.J., Lin T.Y., Hsieh F.J., Chien J.Y., Yeh E.C., Lin W.J., Chen Y.H., Ding K.H., Yang Y., Chi S.C. (2024). Highly efficient capture approach for the identification of diverse inherited retinal disorders. npj Genom. Med..

[2] Michaelides M., Hunt D.M., Moore A.T. (2003). The genetics of inherited macular dystrophies. J. Med. Genet..

[3] Rahman N., Georgiou M., Khan K.N., Michaelides M. (2020). Macular dystrophies: Clinical and imaging features, molecular genetics and therapeutic options. Br. J. Ophthalmol..

[4] Raimondi R., D’Esposito F., Sorrentino T., Tsoutsanis P., De Rosa F.P., Stradiotto E., Barone G., Rizzato A., Allegrini D., Costagliola C. (2023). How to Set Up Genetic Counselling for Inherited Macular Dystrophies: Focus on Genetic Characterization. Int. J. Mol. Sci..

[5] Spedicati B., Morgan A., Pianigiani G., Musante L., Rubinato E., Santin A., Nardone G.G., Faletra F., Girotto G. (2022). Challenging Occam’s Razor: Dual Molecular Diagnoses Explain Entangled Clinical Pictures. Genes.

[6] Lefler W.H., Wadsworth J.A., Sidbury J.B. (1971). Hereditary macular degeneration and amino-aciduria. Am. J. Ophthalmol..

[7] Reichel M.B., Kelsell R.E., Fan J., Gregory C.Y., Evans K., Moore A.T., Hunt D.M., Fitzke F.W., Bird A.C. (1998). Phenotype of a British North Carolina macular dystrophy family linked to chromosome 6q. Br. J. Ophthalmol..

[8] Small K.W., Hermsen V., Gurney N., Fetkenhour C.L., Folk J.C. (1992). North Carolina Macular Dystrophy and Central Areolar Pigment Epithelial Dystrophy: One Family, One Disease. Arch. Ophthalmol..

[9] Small K.W. (1989). North Carolina Macular Dystrophy, Revisited. Ophthalmology.

[10] North Carolina Macular Dystrophy Phenotype in France Maps to the MCDR1 Locus. http://www.emory.edu/molvis/v3/small.

[11] Rabb M.F., Mullen L., Yelchits S., Udar N., Small K.W. (1998). A North Carolina macular dystrophy phenotype in a Belizean family maps to the MCDR1 locus. Am. J. Ophthalmol..

[12] Rohrschneider K., Blankenagel A., Kruse F.E., Fendrich T., Volcker H.E. (1998). Macular function testing in a German pedigree with North Carolina Macular Dystrophy. Retina.

[13] Kim S.J., Woo S.J., Yu H.G. (2006). A Korean Family with an Early-Onset Autosomal Dominant Macular Dystrophy Resembling North Carolina Macular Dystrophy. Korean J. Ophthalmol..

[14] Wu S., Yuan Z., Sun Z., Zhu T., Wei X., Zou X., Sui R. (2022). A novel tandem duplication of PRDM13 in a Chinese family with North Carolina macular dystrophy. Graefe’s Arch. Clin. Exp. Ophthalmol..

[15] Namburi P., Khateb S., Meyer S., Bentovim T., Ratnapriya R., Khramushin A., Swaroop A., Schueler-Furman O., Banin E., Sharon D. (2020). A unique PRDM13-associated variant in a Georgian Jewish family with probable North Carolina macular dystrophy and the possible contribution of a unique CFH variant. Mol. Vis..

[16] Chacon-Camacho O.F., Flores-Lagunes L.L., Small K.W., Udar N., Udar U., Diaz A., Arce-González R., Molina-Garay C., Martínez-Aguilar A., Montes-Almanza L. (2024). A novel PRDM13 gene duplication causing congenital North Carolina macular dystrophy phenotype in a Mexican family. Mol. Vis..

[17] Small K.W., Van de Sompele S., Nuytemans K., Vincent A., Yuregir O.O., Ciloglu E., Sariyildiz C., Rosseel T., Avetisjan J., Udar N. (2021). A novel duplication involving PRDM13 in a Turkish family supports its role in North Carolina macular dystrophy (NCMD/MCDR1). Mol. Vis..

[18] Green D.J., Lenassi E., Manning C.S., McGaughey D., Sharma V., Black G.C., Ellingford J.M., Sergouniotis P.I. (2021). North Carolina macular dystrophy: Phenotypic variability and computational analysis of disease-associated noncoding variants. Investig. Ophthalmol. Vis. Sci..

[19] Tsang S.H., Sharma T. (2018). North Carolina Macular Dystrophy. Atlas of Inherited Retinal Diseases.

[20] Van de Sompele S., Small K.W., Cicekdal M.B., Soriano V.L., D’haene E., Shaya F.S., Agemy S., Van der Snickt T., Rey A.D., Rosseel T. (2022). Multi-omics approach dissects cis-regulatory mechanisms underlying North Carolina macular dystrophy, a retinal enhanceropathy. Am. J. Hum. Genet..

[21] Khurana R.N., Sun X., Pearson E., Yang Z., Harmon J., Goldberg M.F., Zhang K. (2009). A Reappraisal of the Clinical Spectrum of North Carolina Macular Dystrophy. Ophthalmology.

[22] Small K.W., Weber J.L., Hung W.Y., Vance J., Roses A., Pericak-Vance M. (1991). North Carolina macular dystrophy: Exclusion map using RFLPs and microsatellites. Genomics.

[23] Michaelides M., Johnson S., Poulson A., Bradshaw K., Bellmann C., Hunt D.M., Moore A.T. (2003). An Autosomal Dominant Bull’s-Eye Macular Dystrophy (MCDR2) that Maps to the Short Arm of Chromosome 4. Investig. Ophthalmol. Vis. Sci..

[24] Michaelides M., Johnson S., Tekriwal A.K., Holder G.E., Bellmann C., Kinning E., Woodruff G., Trembath R.C., Hunt D.M., Moore A.T. (2003). An early-onset autosomal dominant macular dystrophy (MCDR3) resembling North Carolina macular dystrophy maps to chromosome 5. Investig. Ophthalmol. Vis. Sci..

[25] Michaelides M., Gaillard M.C., Escher P., Tiab L., Bedell M., Borruat F.X., Barthelmes D., Carmona R., Zhang K., White E. (2010). The PROM1 mutation p.R373C causes an autosomal dominant bull’s eye maculopathy associated with rod, rod-cone, and macular dystrophy. Investig. Ophthalmol. Vis. Sci..

[26] Small K.W., DeLuca A.P., Whitmore S.S., Rosenberg T., Silva-Garcia R., Udar N., Puech B., Garcia C.A., Rice T.A., Fishman G.A. (2016). North Carolina Macular Dystrophy Is Caused by Dysregulation of the Retinal Transcription Factor PRDM13. Ophthalmology.

[27] Spedicati B., Santin A., Nardone G.G., Rubinato E., Lenarduzzi S., Graziano C., Garavelli L., Miccoli S., Bigoni S., Morgan A. (2023). The Enigmatic Genetic Landscape of Hereditary Hearing Loss: A Multistep Diagnostic Strategy in the Italian Population. Biomedicines.

[28] Rentzsch P., Witten D., Cooper G.M., Shendure J., Kircher M. (2019). CADD: Predicting the deleteriousness of variants throughout the human genome. Nucleic Acids Res..

[29] Limongelli I., Marini S., Bellazzi R. (2015). PaPI: Pseudo amino acid composition to score human protein-coding variants. BMC Bioinform..

[30] Adzhubei I., Jordan D.M., Sunyaev S.R. (2013). Predicting functional effect of human missense mutations using PolyPhen-2. Curr. Protoc. Hum. Genet..

[31] Ng P.C., Henikoff S. (2003). SIFT: Predicting amino acid changes that affect protein function. Nucleic Acids Res..

[32] Jian X., Boerwinkle E., Liu X. (2014). In silico prediction of splice-altering single nucleotide variants in the human genome. Nucleic Acids Res..

[33] De Coster W., D’Hert S., Schultz D.T., Cruts M., Van Broeckhoven C. (2018). NanoPack: Visualizing and processing long-read sequencing data. Bioinformatics.

[34] Li H. (2018). Minimap2: Pairwise alignment for nucleotide sequences. Bioinformatics.

[35] Smolka M., Paulin L.F., Grochowski C.M., Horner D.W., Mahmoud M., Behera S., Kalef-Ezra E., Gandhi M., Hong K., Pehlivan D. (2024). Detection of mosaic and population-level structural variants with Sniffles2. Nat. Biotechnol..

[36] Belyeu J.R., Chowdhury M., Brown J., Pedersen B.S., Cormier M.J., Quinlan A.R., Layer R.M. (2021). Samplot: A platform for structural variant visual validation and automated filtering. Genome Biol..

[37] Manes G., Joly W., Guignard T., Smirnov V., Berthemy S., Bocquet B., Audo I., Zeitz C., Sahel J., Cazevieille C. (2017). A novel duplication of PRMD13 causes North Carolina macular dystrophy: Overexpression of PRDM13 orthologue in drosophila eye reproduces the human phenotype. Hum. Mol. Genet..

[38] Bowne S.J., Sullivan L.S., Wheaton D.K., Locke K.G., Jones K.D., Koboldt D.C., Fulton R.S., Wilson R.K., Blanton S.H., Birch D.G. (2016). North Carolina macular dystrophy (MCDR1) caused by a novel tandem duplication of the *PRDM13* gene. Mol. Vis..

[39] Zhu Z., Zou H., Li C., Tong B., Zhang C., Xiao J. (2022). The possible pathogenesis of macular caldera in patients with North Carolina macular dystrophy. BMC Ophthalmol..

[40] Tandon M., Barnett C., Taranath D. (2019). Case report: North Carolina macular dystrophy misdiagnosed as congenital ocular toxoplasmosis. Mol. Vis..

[41] Silva R.S., Arno G., Cipriani V., Pontikos N., Defoort-Dhellemmes S., Kalhoro A., Carss K.J., Raymond F.L., Dhaenens C.M., Jensen H. (2019). Unique noncoding variants upstream of PRDM13 are associated with a spectrum of developmental retinal dystrophies including progressive bifocal chorioretinal atrophy. Hum. Mutat..

[42] Small K.W., Wiggins R., Udar N., Silva-Garcia R., Avetisjan J., Vincent A., Shaya F.S. (2022). North Carolina Macular Dystrophy: Long-term Follow-up of the Original Family. Ophthalmol. Retina..

[43] Seo Y., Joo K., Lee J., Diaz A., Jang S., Cherry T.J., Bujakowska K.M., Han J., Woo S.J., Small K.W. (2024). Two novel non-coding single nucleotide variants in the DNase1 hypersensitivity site of *PRDM13* causing North Carolina macular dystrophy in Korea. Mol. Vis..

[44] Goodson N.B., Nahreini J., Randazzo G., Uruena A., Johnson J.E., Brzezinski J.A. (2018). Prdm13 is required for Ebf3+ amacrine cell formation in the retina. Dev. Biol..

[45] Bessodes N., Parain K., Bronchain O., Bellefroid E.J., Perron M. (2017). Prdm13 forms a feedback loop with Ptf1a and is required for glycinergic amacrine cell genesis in the Xenopus Retina. Neural Dev..

[46] Watanabe S., Sanuki R., Sugita Y., Imai W., Yamazaki R., Kozuka T., Ohsuga M., Furukawa T. (2015). Prdm13 Regulates Subtype Specification of Retinal Amacrine Interneurons and Modulates Visual Sensitivity. J. Neurosci..

[47] Small K.W., Tran E.M., Small L., Rao R.C., Shaya F. (2019). Multimodal Imaging and Functional Testing in a North Carolina Macular Disease Family: Toxoplasmosis, Fovea Plana, and Torpedo Maculopathy Are Phenocopies. Ophthalmol. Retin..

[48] Liu Z., Xie Z., Li M. (2024). Comprehensive and deep evaluation of structural variation detection pipelines with third-generation sequencing data. Genome Biol..

[49] Ellingford J.M., Ahn J.W., Bagnall R.D., Baralle D., Barton S., Campbell C., Downes K., Ellard S., Duff-Farrier C., FitzPatrick D.R. (2022). Recommendations for clinical interpretation of variants found in non-coding regions of the genome. Genome Med..

[50] Tomaselli P.J., Rossor A.M., Horga A., Jaunmuktane Z., Carr A., Saveri P., Piscosquito G., Pareyson D., Laura M., Blake J.C. (2017). Mutations in noncoding regions of GJB1 are a major cause of X-linked CMT. Neurology.

[51] Bauwens M., Garanto A., Sangermano R., Naessens S., Weisschuh N., De Zaeytijd J., Khan M., Sadler F., Balikova I., Van Cauwenbergh C. (2019). ABCA4-associated disease as a model for missing heritability in autosomal recessive disorders: Novel noncoding splice, cis-regulatory, structural, and recurrent hypomorphic variants. Genet. Med..

[52] Wright C.F., Quaife N.M., Ramos-Hernández L., Danecek P., Ferla M.P., Samocha K.E., Kaplanis J., Gardner E.J., Eberhardt R.Y., Chao K.R. (2021). Non-coding region variants upstream of MEF2C cause severe developmental disorder through three distinct loss-of-function mechanisms. Am. J. Hum. Genet..

[53] Bruselles A., Mancini C., Chiriatti L., Carvetta M., Baroni M.C., Cappelletti C., Caraffi S.G., Celario M., Ciolfi A., Cordeddu V. (2025). Expanding the mutational spectrum of ReNU syndrome: Insights into 5′ Stem-loop variants. Eur. J. Hum. Genet..

[54] Bakall B., Bryan J.S., Stone E.M., Small K.W. (2021). Choroidal neovascularization in North Carolina Macular Dystrophy responsive to anti-Vascular Endothelial Growth Factor therapy. Retin. Cases Brief Rep..

